# Mitigated suppressive function of regulatory T cells (Treg) upon Th17-inducing cytokines in oligo- and polyarticular Juvenile Idiopathic Arthritis (JIA) patients

**DOI:** 10.1186/s12969-022-00680-z

**Published:** 2022-04-11

**Authors:** Marie-Therese Holzer, Giovanni Almanzar, Robert Woidich, Boris Hügle, Johannes-Peter Haas, Martina Prelog

**Affiliations:** 1 Department of Pediatrics, Pediatric Rheumatology/Special Immunology, University Hospital Wuerzburg, Josef-Schneider-Str. 2, 97080 Wuerzburg, Germany; 2 Department of Internal Medicine III. (Nephrology and Rheumatology With Section Endocrinology), University Hospital Hamburg- Eppendorf, University Hospital Hamburg-Eppendorf, Martinistraße 52, 20246 Hamburg, Germany; 3 German Centre of Pediatric Rheumatology, Gehfeldstraße 24, 82467 Garmisch-Partenkirchen, Germany

**Keywords:** Treg, Th17, JIA, IL-17A-inhibition, Suppressive function, T cell plasticity

## Abstract

**Background:**

The plasticity of T helper-17 (Th17) and regulatory T (Treg) cells may be a clue to pathogenesis of Juvenile Idiopathic Arthritis (JIA). It is still unclear, whether targeted suppression of Interleukin (IL)-17 is able to influence regulatory function of Treg to control pro-inflammatory effectors in JIA. This study aimed to assess the effect of a Th17-stimulating cytokine environment and of IL-17A-inhibition on phenotype plasticity and suppressive function of Treg derived from JIA patients.

**Methods:**

Th17 and Treg characteristics of CD4^+^ helper T cells were investigated in blood samples of JIA patients with oligo- and polyarticular pattern and healthy controls (HC). Isolated CD4^+^CD25^+^CD127^−^ cells defined as Treg were cultivated with Th17-inducing cytokine environment as well as with IL-17A-inhibitors and analyzed for plasticity of phenotype by flow cytometry. Furthermore, inhibitory function of Treg on autologous effectors after cultivation with these stimuli was determined by suppression assays.

**Results:**

Our findings demonstrated significantly elevated proportions of Th17 and Th17-like Treg in JIA compared to HC. After incubation with Th17-inducing stimuli, increased FoxP3 expression in separated Treg in JIA and an impaired suppressive capacity in JIA and HC were found. Blockade of IL-17A resulted in adjustment of FoxP3-expression in JIA to proportions found in controls and in regular suppressive function.

**Conclusions:**

Our results demonstrate an induction of FoxP3 expressing Treg by Th17-inducing cytokines with concomitant mitigated suppressive function. In contrast, specific IL-17A blockade maintains suppressive Treg function and adjusted FoxP3-expression in JIA to levels found in controls. These findings may help to provide experimental evidence for the successful clinical use of IL-17A inhibition in JIA patients.

**Supplementary Information:**

The online version contains supplementary material available at 10.1186/s12969-022-00680-z.

## Background

T cells play a central role in the pathogenesis of Juvenile Idiopathic Arthritis (JIA) with activated T cells accumulating in inflamed joints [1]. Research has been focusing on the balance of Th17 cells and regulatory T cells (Treg) with so far inconclusive results. Both subpopulations were elevated in the peripheral blood of children with active JIA [2]. Other studies found lower Treg frequencies in the peripheral blood of JIA compared to healthy individuals and a reciprocal relationship between Th17 and Treg cells as well as of specific transcription factors RORγt and FoxP3, respectively, in synovial fluid of inflamed joints [3–5]. Treg themselves are usually divided into naturally occurring/thymic and induced/peripheral Treg with the latter developing out of naive T cells by gaining FoxP3 expression via CD28-costimulation and stimulation with IL-2 and TGFβ [6].

FoxP3 as one of the most important Treg markers is discussed to reflect stability and suppressive function of Treg [7, 6]. Contentious findings show an unstable FoxP3-expression in inflammatory environments driving Treg to convert into proinflammatory cells [8, 9] whereas others showed a stable FoxP3-lineage of Treg even in infectious and autoimmune environments [10]. Moreover, FoxP3 transcription is persistently increased during inflammation which is proposed as a possible mechanism of promoting Treg features for immune regulation [11].

A close relationship exists between Th17 and Treg. Treg differentiating into Th17 cells in presence of IL-6 and into IL-17-producing FoxP3^+^ T cells in the synovia of active autoimmune arthritis [12, 13] encourage the ongoing debate on plasticity of these two cell groups. Th17-like Treg and Treg expressing CD161, a characteristic marker for Th17/Th1 switching cell lines, seem to maintain suppressive function unless Treg start to produce IL-17 through activation by a pro-inflammatory cytokine environment [14–16].

Serum and synovial IL-17 levels were elevated in JIA patients and IL-17 significantly contributed to the pro-inflammatory conditions in specific JIA subtypes [17]. Secukinumab is a human monoclonal antibody directed against IL-17A. Similar to the positive effect in psoriatic arthritis and ankylosing arthritis in adults, the blockade of IL-17 may be useful especially in the JIA subtypes with high IL-17 levels like psoriatic and enthesitis-related arthritis [18]. So far, an approved pediatric indication for Secukinumab is psoriasis, phase III studies for enthesitis-associated and psoriatic JIA are ongoing.

To this date, only frequency and phenotype of Treg and Th17 have been studied in JIA. It remains unclear whether the targeted suppression of IL-17 is able to positively influence the regulatory function of Treg to control pro-inflammatory effectors in JIA and whether FoxP3 is up-regulated in this process. Thus, this study aimed to assess the influence of a Th17-stimulating cytokine environment and of inhibition of IL-17A not only on the plasticity of the Treg phenotype but also on the suppressive function of Treg derived from JIA patients as a potential add-on effect of IL-17A-targeted therapy.

## Methods

Blood samples of 14 patients diagnosed with extended (*n* = 9) or persisting oligoarticular (*n* = 1) and seronegative polyarticular (*n* = 4) JIA according to ILAR-classification [19] were collected at the German Center for Pediatric and Adolescent Rheumatology, Garmisch-Partenkirchen. Due to known differences in pathophysiology [20], other JIA subtypes like enthesitis-associated, psoriatic or systemic JIA were excluded. Blood samples of 10 immunologically healthy controls (HC) were obtained at the University Hospital Wuerzburg. Exclusion criteria were malignancies, hematological diseases, monogenetic syndromes, severe infection requiring medical support in the past 3 months prior to inclusion, severe allergies and status post transplantation. No autoimmune disorders were reported in HC or first line relatives. The study was performed as an explorative prospective controlled cohort study according to the principles of the Declaration of Helsinki 2013 and was approved by the local ethics committee at the University Hospital Wuerzburg (protocol No. 239/10).

First, a characterization of CD4^+^ T helper (Th) cells was performed. Peripheral blood mononuclear cells (PBMC) were obtained from venous blood samples with density gradient centrifugation with FicoLite-H (Linaris blue GmbH, Wertheim, Germany) according to the standard laboratory protocol, stored until use in liquid nitrogen after preparation with 10% dimethyl sulfoxide (DMSO, Carl Roth GmbH, Karlsruhe, Germany) and analyzed with flow cytometry by staining after four hours of stimulation with Phorbol 12-myristate 13-acetate (PMA), Ionomycin and Brefeldin A (Sigma-Aldrich, St. Louis, USA) for surface molecules (CXCR3, CCR6, CD127, CD161, CD25, CD27, CD4, and zombie dye to exclude dead cells, all purchased from BioLegend, San Diego, USA) and with intracellular markers of Th17 and Treg cells (FoxP3, RORγt, IL-17, BD Biosciences, Franklin Lakes, USA and BioLegend) as described previously [21]. Differences between JIA and HC were assessed with BD FACS Diva Software Version 6.1.3.

To analyze the influence of different cytokines on Treg phenotype a magnetic-assisted isolation of CD4^+^CD25^+^CD127^−^ cells defined as isolated Treg was performed with Miltenyi Biotec MACS Treg Kit II (Bergisch-Gladbach, Germany) as described earlier [21].

Isolated Treg were incubated for 5 days at 37 °C and 5% CO_2_ as follows: Culture medium RPMI-1640 (Sigma-Aldrich) with 1% Penicillin/Streptomycin (Carl Roth GmbH) and 10% fetal bovine serum (Biochrom AG, Berlin, Germany) was used as negative control. Unspecific T cell receptor stimulation with anti-CD3 1 μg/ml (antibody clone OKT3) and anti-CD28 0.5 μg/ml (antibody clone CD28.2) (BioLegend) served as positive control. Treg were cultured under the following conditions: for IL-17 inhibition by 10 μg/ml anti-IL-17A (research-use-only antibody, clone eBio64CAP17, Invitrogen Thermo Fisher, Carlsbad, USA) or by 10 μg/ml Secukinumab (Novartis, Basel, Switzerland) with concomitant anti-CD3/anti-CD28 stimulation, respectively. A Th17-polarizing cytokine stimulation was simulated by IL-1β (10 ng/ml), IL-6 (20 ng/ml), IL-23 (10 μg/ml) and TGFβ (5 ng/ml) (all purchased from BioLegend) and concomitant anti-CD3/anti-CD28 stimulation. After 5 days cell culture, Treg were stained and assessed by flow cytometry as described above. Differences in cell-death rates could be excluded by Zombie staining of dead cells.

The influence of the various stimuli on the suppressive function of Treg on autologous PBMC was assessed utilizing dilution of Carboxyfluorescein-succinimidyl-ester (CFSE, BioLegend) for detection of proliferated PBMC in suppression assays as described previously [21]. PBMC and Treg were co-cultured at equal amounts of cells in Th17-inducing cytokine conditions or under blockade of IL-17A as well as in negative and positive control conditions as described above. Inhibition of PBMC by Treg in % was determined as 1 minus the fraction of proliferation of PBMC in 1:1 co-culture with Treg and the proliferation of PBMC without Treg multiplied by 100.

After testing for distribution (Shapiro-Wilks-test), Mann–Whitney-U-test was applied for non-parametric, continuous independent variables (statistics software SPSS version 24.0, IBM statistics, Chicago, USA). A *p*-value less than 0.05 was considered statistically significant. Correction of type I error due to multiple comparisons was done with Bonferroni correction for each experiment, respectively.

## Results

Demographics and clinical parameters of the study group are shown in Table 1. Thirteen patients (92.9%) were on medication and presented to be in laboratory remission according to C-reactive protein, erythrocyte sedimentation rate, total leucocyte, lymphocyte and thrombocyte counts (all within normal age-range) with clinically active joints in only four patients.

**Table 1:** Characteristics of the study population (A) and clinical data of Juvenile Idiopathic Arthritis patients (B)

**(A) Study population**	**JIA**	**HC**
**Number of samples**	14	10
**Numbers of male (%)/** **female (%)**	6 (42.9%)/ 8 (57.1%)	2 (20%)/ 8 (80%)
**Age at time of inclusion (years)**	16.1 ± 5.0 (16; 8–27)	23.4 ± 1.69 (23.5; 19–25)
**Age at time of diagnosis (years)**	5 ± 4.1 (3.5; 1–15)	-
**Disease duration (years)**	11.1 ± 6.0 (12.5; 1–24)	-
**(B) Clinical Data in JIA**		
**Medication**	13/92.9%	
**Nonsteroidal anti-inflammatory drug** (in therapeutic dosages)	7/50%	
**Methotrexate** (at < 15 mg/m^2^/week)	7/50%	
**Glucocorticoids** (at < 0.1 mg/kg/d)	3/21.4%	
**Biologicals** (in therapeutic dosages)	10/71.4%	
	Tocilizumab 1/7.1%	
	Adalimumab 4/28.6%	
	Abatacept 2/14.3%	
	Etanercept 3/21.4%	
**Mycophenolate Mofetil**	2/14.3%	
**Antinuclear antibodies positive**	11/78.6%	
**Rheumatoid factor positive**	0/0%	
**C-reactive protein (mg/dl)**	0.2 ± 0.3 (0.0; 0–0.9)	
**Erythrocyte sedimentation rate (mm/h)**	11.7 ± 10.7 (6.5; 2–35)	
**Leukocytes (× 10**^**3**^** per μl)**	7.0 ± 2.3 (6.7; 2.4–11.5)	
**Lymphocytes (G/l)**	2.6 ± 0.8 (2.6; 1.3–4.3)	
**Lymphocytes (%)**	39.9 ± 10.6 (39.5; 13.8–64)	
**Thrombocytes (× 10**^**3**^** per μl)**	231.3 ± 54.6 (232; 138–327)	
**Immunoglobulin G (mg/dl**)	912.9 ± 353.1 (992; 133–1309)	
**Active joints**	4/28.5% ACR active joints if positive: 1; 3; 3; 4	

Characteristics of the study population are shown in (A). Values are given in absolute numbers (percentages) or as mean ± SD (median; minimum to maximum). *HC* healthy controls, *JIA* Juvenile Idiopathic Arthritis

Clinical data of Juvenile Idiopathic Arthritis (JIA) patients are gathered in (B). Number of patients = 14. Values are given in absolute numbers/percentages or as mean ± SD (median; minimum—maximum). *ACR* American College of Rheumatology

T cell characterization (analysis of PBMC cells) revealed elevated proportions of CD4^+^ and Treg cells expressing Th17 markers in JIA patients compared to HC (Table 2). Significantly higher proportions of CCR6- and CD161-co-expressing CD4^+^ helper T cells were found in JIA compared to HC (median 0.7% vs. 0.1%, *p* = 0.002). CXCR3^+^, a characteristic chemokine receptor of Th1, was not significantly different between JIA and HC. Proportions of IL-17 producing CD4^+^ (median 0.8% vs. 0.3%) and FoxP3^+^CD4^+^ cells (median 2.85% vs. 0.6%) were higher in JIA compared to HC (*p* = 0.002, respectively) whereas cells characterizing the natural occurring Treg phenotype (FoxP3^+^, CD25^+^, CD127^−^) were on a similar level as in HC.

**Table 2:** Comparison of Th17- and regulatory T cells-specific markers in peripheral CD4^+^ helper T cells

Gate	Subpopulation	JIA	HC	*p*-value
**CD4**^**+**^	**CCR6**^**+**^	8.01 ± 4.35 (7.25; 1.40–16.60)	1.80 ± 0.90 (1.60; 0.90–3.20)	0.011
**CD4**^**+**^	**CD161**^**+**^	7.06 ± 4.70 (6.30; 1.00–14.40)	1.98 ± 1.19 (2.70; 0.40–2.90)	0.011
**CD4**^**+**^	**CCR6**^**+**^ **CD161**^**+**^	0.85 ± 0.60 (0.70; 0.20–1.80)	0.08 ± 0.08 (0.10; 0.00–0.20)	0.002*
**CD4**^**+**^	**RORγt**^**+**^	0.58 ± 0.43 (0.45; 0.30–1.60)	1.06 ± 0.53 (1.00; 0.50–1.70)	0.045
**CD4**^**+**^	**IL-17**^**+**^	1.10 ± 0.79 (0.80; 0.50–2.90)	0.24 ± 0.09 (0.30; 0.10–0.30)	0.002*
**CD4**^**+**^**RORγt**^**+**^	**IL-17**^**+**^	7.48 ± 3.74 (7.30; 2.20–13.10)	2.54 ± 2.46 (1.00; 0.60–6.00)	0.019
**CD4**^**+**^	**FoxP3**^**+**^	3.59 ± 1.82 (3.25; 1.70–6.50)	2.26 ± 1.01 (2.30; 1.10–3.30)	0.171
**CD4**^**+**^**FoxP3**^**+**^	**IL-17**^**+**^	3.10 ± 1.43 (2.85; 1.20–5.60)	0.62 ± 0.23 (0.60; 0.40–1.00)	0.002*
**CD4**^**+**^**FoxP3**^**+**^	**CD25**^**+**^ **CD127**^**−**^	24.94 ± 10.79 (24.45; 8.30–41.20)	24.00 ± 4.83 (22.40; 18.50–30.80)	1.000

Peripheral blood mononuclear cells were assessed for Th17- and regulatory T cells (Treg)-characteristic markers by flow cytometry. Values of 5 healthy controls (HC) and 8 patients with Juvenile Idiopathic Arthritis (JIA) were compared with Mann–Whitney-U-test. Data are presented as percentages in mean ± standard deviation (median; minimum—maximum). After correction for multiple comparison with Bonferroni correction the results marked with * remained statistically significant. Elevated proportions of Th17/Th1 switching cells as well as CD4^+^ and CD4^+^ FoxP3^+^ cells producing IL-17 were assessed in JIA patients compared to HC

**Fig. 1:**
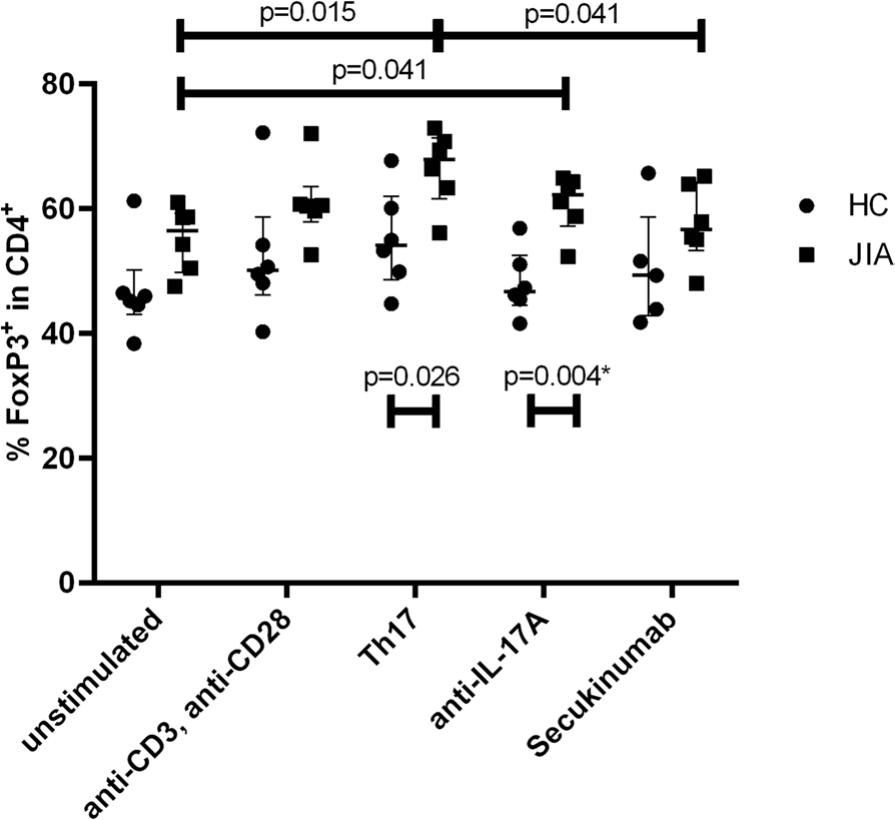
Influence of different stimuli on phenotype of isolated regulatory T cells. The influence of different stimuli on isolated Treg was assessed by flowcytometry after 5 days of culture and values of FoxP3^+^CD4^+^ cells in isolated regulatory T cells (Treg) are shown in percentage (median and interquartile ranges). As stimuli were used: unstimulated = none; anti-CD3/CD28 as positive control; Th17 = anti-CD3/CD28 + IL-1β + IL-6 + IL-23 + TGFβ; anti-IL17A = anti-CD3/CD28 + laboratory IL-17A-antibody; Secukinumab = anti-CD3/28 + Secukinumab. *N* = 6 for healthy controls (HC) and Juvenile Idiopathic Arthritis (JIA) each, except for Secukinumab in HC (here *n* = 5). Groups as well as stimuli were compared for differences with Mann–Whitney-U-test. Whilst elevated level of FoxP3^+^CD4^+^ cells could be seen after Th17-inducing stimuli in HC and JIA and furthermore in every stimulus for JIA compared to HC (with smallest difference in Secukinumab, *p* > 0.2), correction for multiple comparison with Bonferroni correction showed significant difference for FoxP3^+^CD4^+^ cells between JIA and HC after anti-IL-17A cultivation (*p* = 0.004, marked with *)

After 5 days of cultivation of isolated CD4^+^CD25^+^CD127^−^ Treg, an increase of FoxP3^+^ helper T cells (median 67.9%) in Th17 environment was found compared to HC (54.2%) (*p* = 0.026) and compared to unstimulated control (56.5%) (*p* = 0.015) (Fig. 1). In contrast to IL-17A blockade by the research-use-only antibody, where still a significant difference in FoxP3-expression was seen between JIA and HC (*p* = 0.004), in vitro treatment with Secukinumab adjusted FoxP3-expression of JIA-derived Treg to normal levels found in HC (56.7% vs. 49.3%; *p* = 0.247). There was no significant difference in the proportions of IL-17^+^ in FoxP3^+^CD4^+^ cells after different Th17-stimulatory and IL-17A-inhibitory treatments in cultivated Treg (data not shown).

**Fig. 2:**
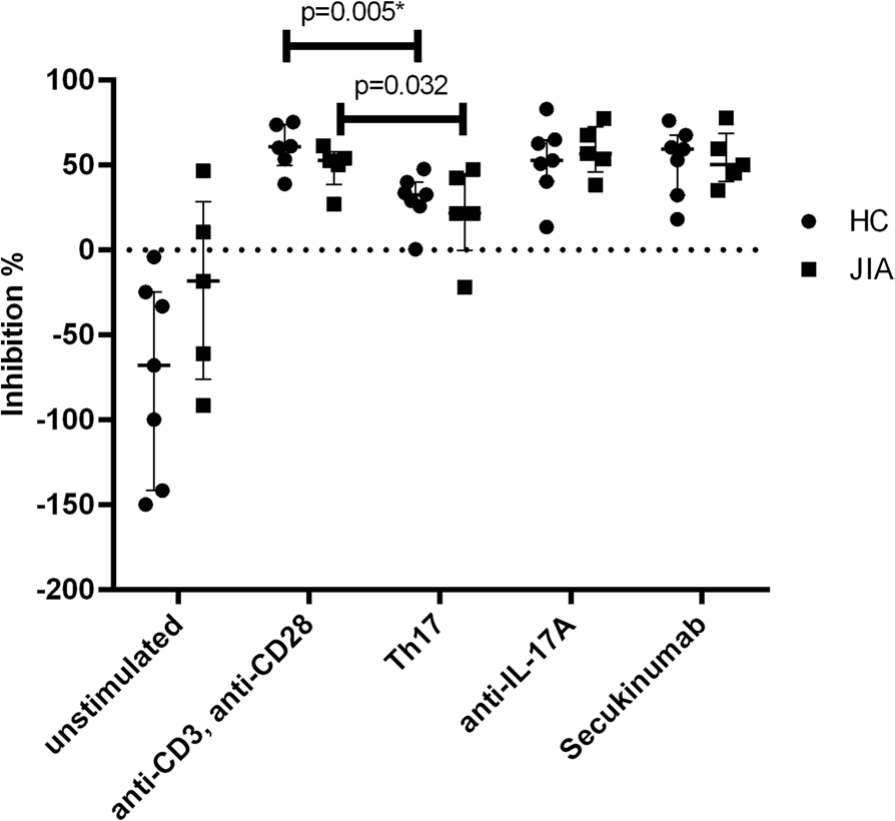
Influence of stimuli on suppressive function of regulatory T cells on peripheral blood mononuclear cells. Isolated regulatory T cells (Treg) and autologous Peripheral blood mononuclear cells (PBMC) were co-cultured in different stimuli. The suppressive function of Treg on PBMC as inhibition in % was calculated as 1-(proliferation of PBMC in 1:1 Co-Culture with isolated Treg × 100) ÷ (proliferation of PBMC in 1:0 Culture) using Carboxyfluorescein-succinimidyl-ester-(CFSE)-assay, assessed by flowcytometry after 5 days of culture. Values are shown in percentage (median and interquartile ranges). As stimuli were used: unstimulated = none; anti-CD3/CD28 as positive control; Th17 = anti-CD3/CD28 + IL-1β + IL-6 + IL-23 + TGFβ; anti-IL17A = anti-CD3/CD28 + laboratory IL-17A-antibody; Secukinumab = anti-CD3/28 + Secukinumab. *N* = 7 for each stimulus in healthy controls (HC), except for anti-CD3/anti-CD28 (*n* = 6) and *n* = 5 for each stimulus in Juvenile Idiopathic Arthritis (JIA). For stimuli comparison all stimuli were compared with anti-CD3/anti-CD28, which served as positive control. Groups as well as stimuli were compared for differences with Mann–Whitney-U-test. Mitigated inhibitory function was seen in Th17 environment in both groups. Difference between positive control and Th17-inducing cytokine environment in HC was significant (*p* = 0.005, marked with *) after Bonferroni correction was applied. Difference between negative control (= unstimulated) and positive control in HC was statistically significant as well (*p* = 0.001, not marked)

An analysis of the suppressive effect of isolated Treg on co-cultured autologous PBMC in different cytokine environment was performed to determine the influence of the stimuli on the functional capacity of the isolated Treg. Inhibition of PBMC proliferation by Treg was mitigated compared to the positive control when cultured with additional Th17-inducing cytokines both in JIA (52.5% vs. 21.5%; *p* = 0.032) and HC (60.5% vs. 32.5%; *p* = 0.005). IL-17A blockade with the research-use-only antibody as well as in vitro inhibition of IL-17A by Secukinumab resulted in steady and almost equal inhibition of PBMC proliferation compared to positive control in both JIA (50.0% vs. 52.5%; *p* = 1.00) and HC (59.1% vs. 60.5%; *p* = 0.534) (Fig. 2).

## Discussion

Our findings demonstrate significantly elevated proportions of Th17 cells and Th17-like Treg in peripheral CD4^+^ helper T cells derived from JIA patients compared to HC. A shift towards Th17 and Th17-like Treg has been described by others [3, 12, 17, 18]. The elevated proportions of IL-17 producing FoxP3^+^CD4^+^ cells found in our cohort, may reflect either a specialization of Treg on Th17 cells in JIA as discussed by Campbell et al. for autoimmune diseases in general [8] or echo an increased plasticity of Treg towards the proinflammatory Th17 type [14]. Additionally, IL-17 producing cells derived from former Treg are discussed to be especially pathogenic in arthritic environment, e.g. they seem to be even more osteoclastogenic than for example Th17 cells from naïve CD4^+^ cells are [12]. Opposed to these findings of elevated Th17 and Th17-like Treg we discovered lower proportions of RORγt in JIA patients compared to HC. This might be due to a reciprocal relation to the transcription factor FoxP3 which is known to suppress the expression of RORγt [4]. Furthermore, CCR6 and CD161 were introduced as markers of Th17/Th1 switching cells elevated in JIA compared to HC. Elevated Th17/Th1 switching cells have also been described earlier by Nistala et al. in inflamed joints of JIA patients [22].

Our in vitro results provide experimental evidence of plasticity of JIA-derived Treg with FoxP3-induction by Th17-stimulating cytokines mimicking in vivo inflammation. Whereas one study revealed the loss of FoxP3 in the presence of IL-6 [8], others demonstrated stable FoxP3 expression in Treg in an inflammatory environment [10] and furthermore, an increased FoxP3 expression in T cells by cell activation and by TGFβ [6]. The high sensitivity of JIA-derived Treg for Th17-inducing stimuli seen in higher level of FoxP3^+^ cells compared to HC may corroborate the supposed idea of an involvement of Th17-mediated pathways in the pathogenesis of many T cell-mediated autoimmune diseases [8, 14], such as JIA.

In contrast to increased FoxP3 expression in JIA in isolated Treg after 5 days of incubation in cell culture media enriched with Th17-inducing stimuli, specific in vitro blockade of IL-17A resulted in adjustment of FoxP3-expression to normal proportions as found in controls which might underline one mechanism of clinical effectiveness of IL-17 blockade in rheumatic diseases.

Specific IL-17A blockade with Secukinumab seems to be more efficient to allow adjustment to normal levels of FoxP3 expression than IL-17A blockade by the research-use-only antibody. Although there is no literature yet, it might be explained by unknown mechanistic differences between the inhibitory function of Secukinumab and of the research-use-only antibody.

Increased level of FoxP3 in the case of Th17-inducing stimuli may furthermore not be an expression of enrichment of naturally occurring Treg but more likely a hint of CD4^+^ T cell activation or induction of the induced Treg phenotype [11, 23]. Although our experiments are based on peripherally circulating Treg cells, simulation of an inflammatory environment (e.g. as seen in inflamed joints) results in a significant reduction of inhibitory capacities of separated Treg on autologous PBMC. Our functional assays may support the finding that the phenotypically characterized FoxP3^+^ Treg are rather induced Treg which have at least partly lost or even not skilled their suppressive functions. Further on, an increased FoxP3-expression concomitant with mitigated inhibition may also be due to an increased resistance of effector cells towards Treg rather than to an impaired Treg function [16, 24]. Such lower sensitivity of different effector cells towards Treg suppression was discussed in other experiments of our research group of lymphocytes derived from patients with scleroderma [25].

The limitations of this study include the high heterogeneity of JIA disease itself, of therapy and frequency of remittent disease and inflammatory activity. To overcome these problems, only oligo- and polyarticular subtypes and only JIA patients in laboratory remission or with very low clinical disease activity in four patients were included in the study. Enthesitis-associated, psoriatic and systemic JIA were not included due to the known differences in pathophysiology and presentation [20, 26]. The highly standardized experimental protocol on separated Treg and culture in a pro-inflammatory and an anti-inflammatory environment allows to analyze the Treg function in-depth in a small sample of JIA patients while still keeping in mind the intrinsic heterogeneity of JIA subtypes. Possibly, findings would have been more jutting out using lymphocytes obtained during acute disease flare or from inflamed joints of therapy-naive recently diagnosed JIA patients. Furthermore it is important to mention that aging changes the cell subtypes of the immune system, therefore our study represents the examined adolescent group, not young children [27]. Additional experiments regarding epigenetic factors of FoxP3 stability as well as changes at the transcriptome level as performed in scleroderma [21, 28] would have been interesting to assess but were not possible due to relatively low cell counts in volume-restricted blood samples in JIA patients.

## Conclusion

In conclusion, our study supports findings of a shift towards Th17 and Th17-like Treg in JIA, underlining the importance of the Th17 pathway in the pathophysiology of JIA. Furthermore, our experiments show an increased FoxP3-expression in Treg by Th17-inducing cytokines with concomitant mitigated suppressive function. Whereas JIA Treg showed higher FoxP3-expression suggesting stronger cell activation by inflammatory stimuli, specific IL-17A blockade with Secukinumab lead to adjusted FoxP3 levels and suppressive function as found in controls. This may display a potential add-on benefit of specific IL-17A blockade in JIA patients and therefore may help to provide supporting experimental data for the clinical use of IL-17A inhibition in JIA patients.

## Supplementary Information

**Additional file 1.**

## Data Availability

The datasets used and/or analysed during the current study are available from the corresponding author on reasonable request.

## Abbreviations

CCR: Chemokine Receptor
CD: Cluster of Differentiation
CFSE: Carboxyfluorescein-succinimidyl-ester
CXCR3: CXC-Motive-Chemokine-Receptor 3
DMSO: Dimethyl Sulfoxide
Et. al.: Et alii/aliae/alia (and others)
FACS: Flurescence-Activated Cell Sorting
FoxP3: Forkhead-Box-Protein 3
HC: Healthy Control
IL: Interleukin
ILAR: International League of Associations for Rheumatology
JIA: Juvenile Idiopathic Arthritis
MACS: Magnetic-Activated Cell Sorting
PBMC: Peripheral Blood Mononuclear Cells
PMA: Phorbol 12-myristate 13-acetate
RORγt: RAR-related orphan receptor γt
SD: Standard deviation
TGF: Transforming growth factor
Th1/17: T helper cells of Th1/17 subtype
Treg: Regulatory T cells
