# Supplementary material for: Mitigated suppressive function of regulatory T cells (Treg) upon Th17-inducing cytokines in oligo- and polyarticular Juvenile Idiopathic Arthritis (JIA) patients
Source: Pediatr Rheumatol Online J. 2022 Apr 11;20:26. doi: 10.1186/s12969-022-00680-z (PMC8996624; doi:10.1186/s12969-022-00680-z)
Supplement: Supplementary file 1 — Additional file 1. [file 12969_2022_680_MOESM1_ESM.docx]

**Supplementary Figure 1: Purity measurements after isolation of regulatory T cells by magnetic-assisted cell isolation in a representative Healthy Control (HC) and a Juvenile Idiopathic Arthritis (JIA) patient**

| **HC** | **JIA** |
| --- | --- |
| 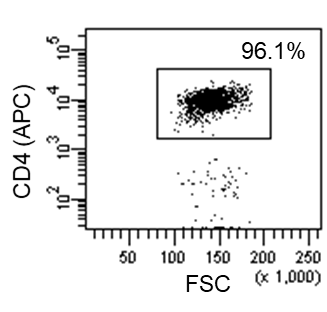 | 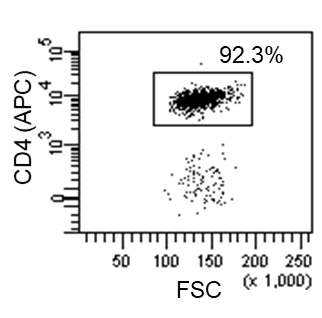 |
| 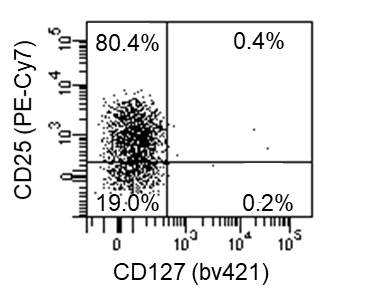 | 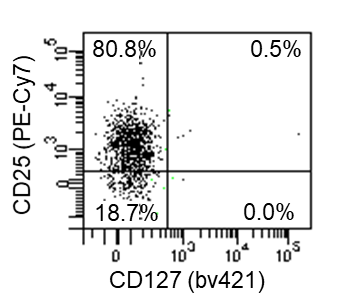 |

**Supplementary Figure 2:** **Representative flow cytometry plot showing gating strategy after cultivation of isolated regulatory T cells with anti-CD3, anti-CD28 stimulation in a Juvenile Idiopathic Arthritis patient**

| **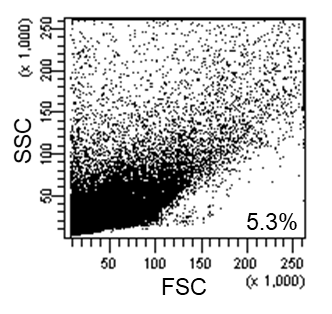** | Gating of Lymphocytes in all events: Sideward (SSC) versus Forward (FSC) Scatter **↓** |
| --- | --- |
| 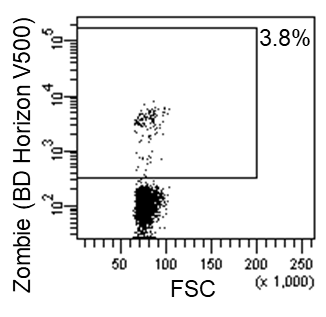 | Gating of Zombie in Lymphocytes (non-viable cells) **↓** |
| 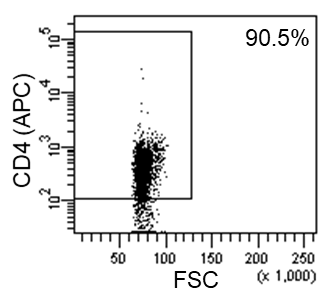 | Gating of CD4^+^ cells in Non-Zombie-Lymphocytes **↓** |
| 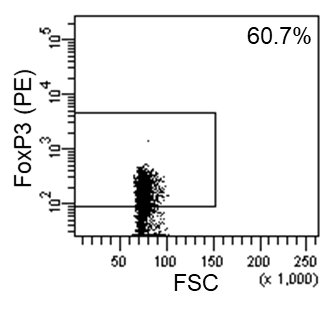 | Gating of FoxP3^+^ cells in CD4^+^-Non-Zombie-Lymphocytes **↓** |
| 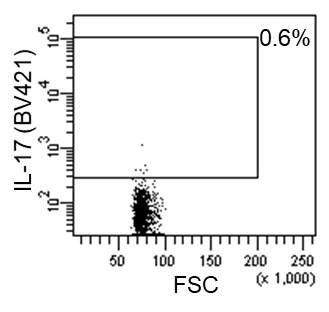 | Gating of IL-17^+^ cells in FoxP3^+^CD4^+^-Non-Zombie-Lymphocytes |

**Supplementary Table 1: Representative dot plots for T helper cell characterization in one Juvenile Idiopathic Arthritis (JIA) patient and one Healthy Control (HC)**

| **Gate** | **Sub­population** | **JIA** | **HC** |
| --- | --- | --- | --- |
| **CD4^+^** | **CCR6^+^** | 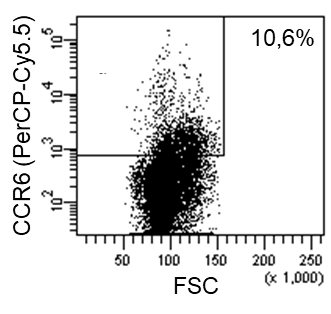 | 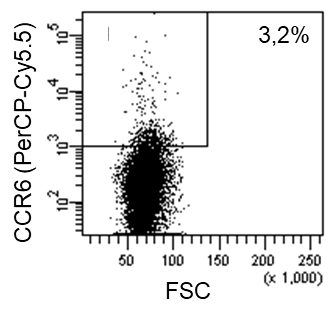 |
| **CD4^+^** | **CD161^+^** | 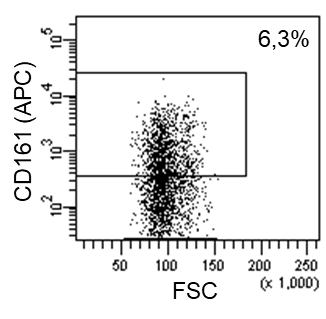 | 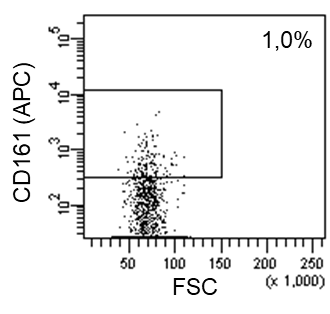 |
| **CD4^+^** | **CCR6^+^ CD161^+^** | 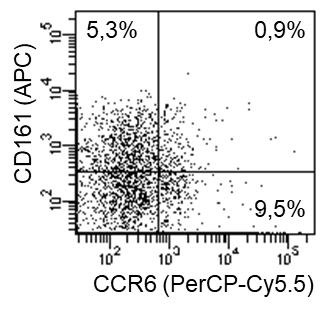 | 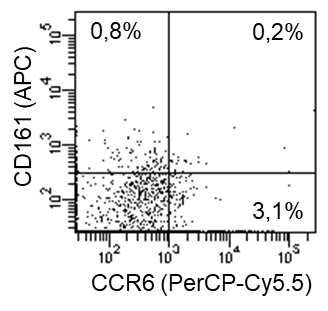 |
| **CD4^+^** | **RORγt^+^** | 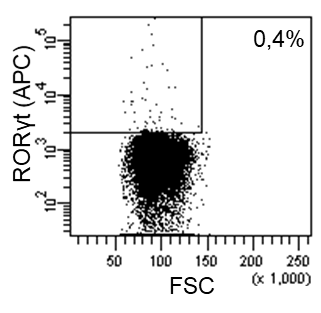 | 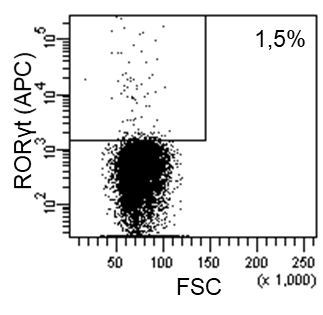 |
| **CD4^+^** | **IL-17^+^** | 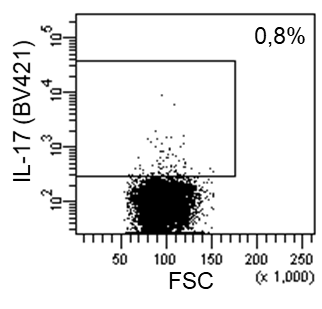 | 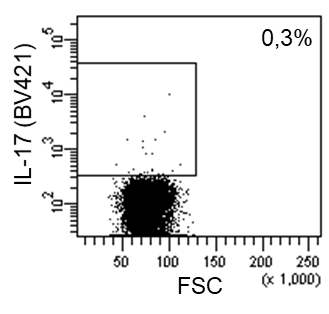 |
| **CD4^+^RORγt^+^** | **IL-17^+^** | 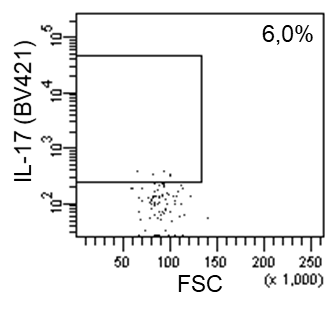 | 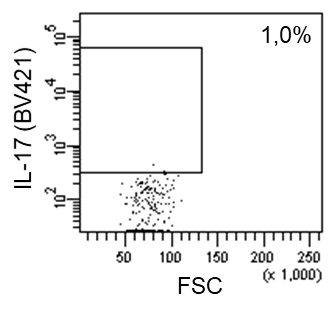 |
| **CD4^+^** | **FoxP3^+^** | 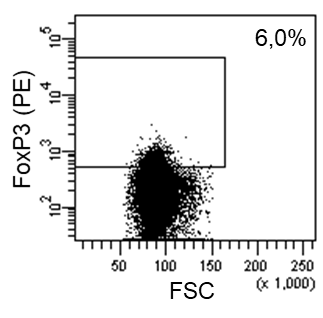 | 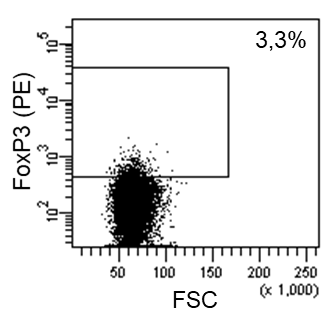 |
| **CD4^+^FoxP3^+^** | **IL-17^+^** | 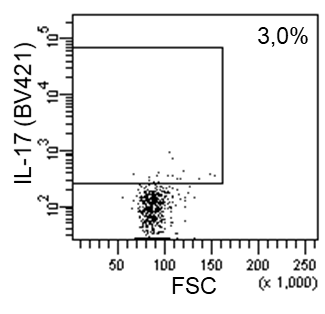 | 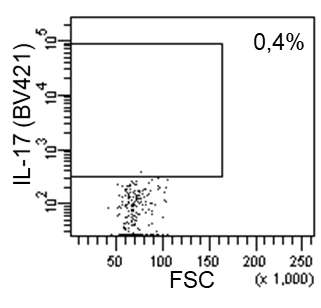 |
| **CD4^+^FoxP3^+^** | **CD25^+^ CD127^-^** | 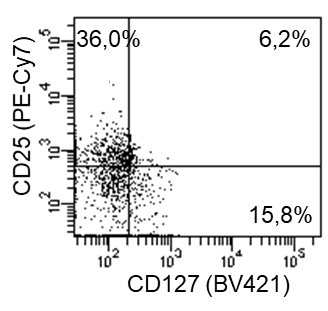 | 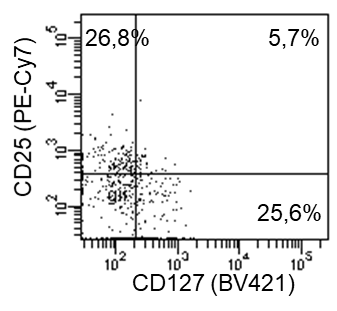 |

**Supplementary Table 2: Example of gating strategy in regulatory T cell (Treg) characterization in one Juvenile Idiopathic Arthritis patient in unstimulated (negative) and in anti-CD3/anti-CD28 (positive) control**

| **Gate** | **unstimulated** | **anti-CD3/anti-CD28** |
| --- | --- | --- |
| **Lymphocytes**  **↓** | **^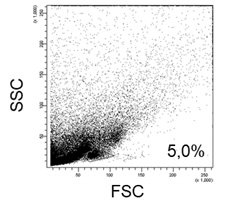^** | 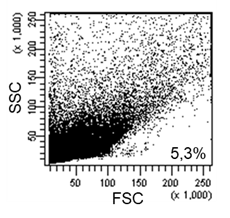 |
| **Zombie in Lymphocytes**  **↓** | 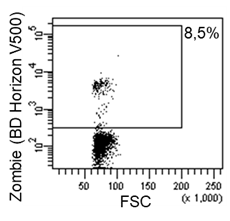 | 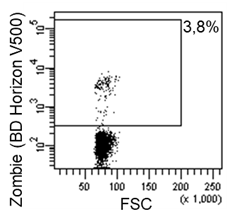 |
| **CD4^+^ in non-Zombie-Lymphocytes**  **↓** | 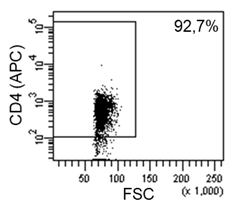 | 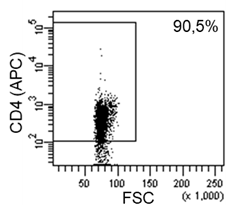 |
| **FoxP3^+^ in CD4^+^in non-Zombie-Lymphocytes** | 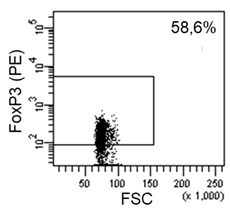 | 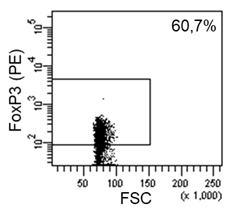 |

CD4^+^CD25^+^CD127^-^ Treg were isolated out of peripheral blood mononuclear cells (PBMCs) by magnetic-assisted isolation and cultured with different stimuli. The gating strategy in FACS Diva Software is shown.

**Supplementary Table 3: Representative plots for suppression assay in one Juvenile Idiopathic Arthritis (JIA) patient and one Healthy Control (HC)**

| **Stimulus** | **JIA** | | **HC** | |
| --- | --- | --- | --- | --- |
|  | **0 Treg : 1 PBMC** | **1 Treg : 1 PBMC** | **0 Treg : 1 PBMC** | **1 Treg : 1 PBMC** |
| **unstimulated** | 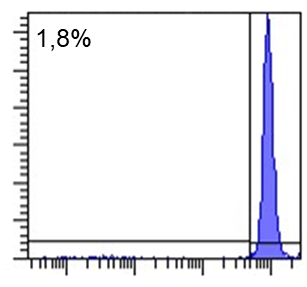 | 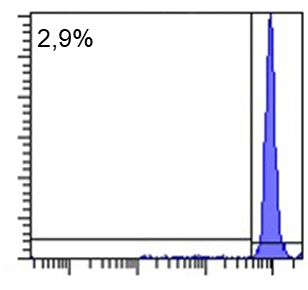 | 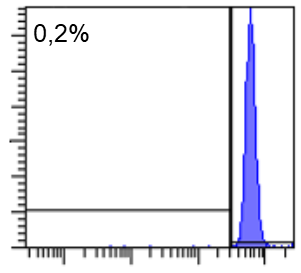 | 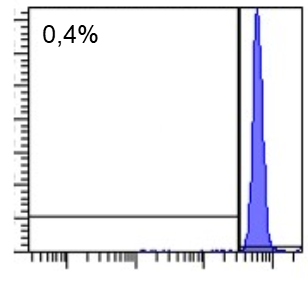 |
| **anti-CD3, anti-CD28** | 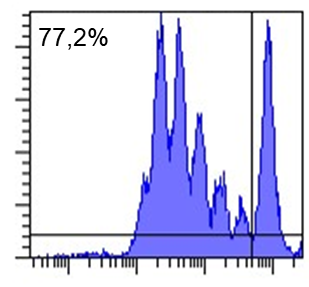 | 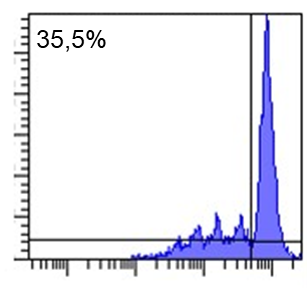 | 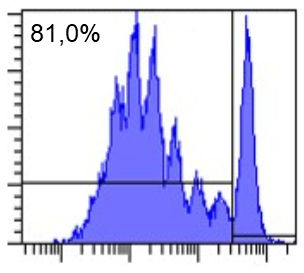 | 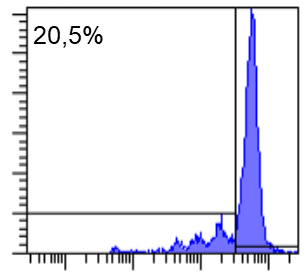 |
| **Th17** | 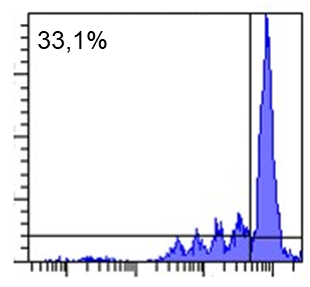 | 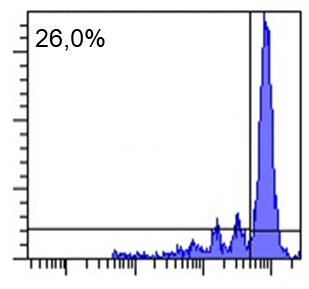 | 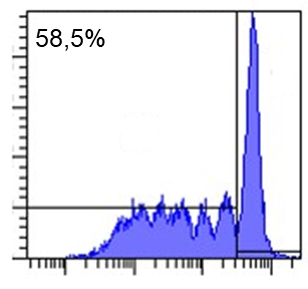 | 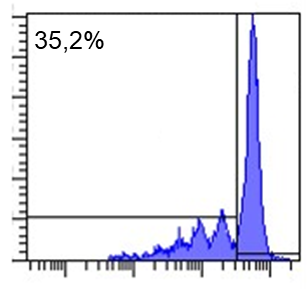 |
| **anti-IL-17** | 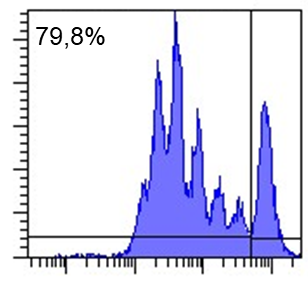 | 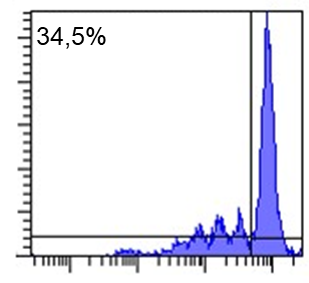 | 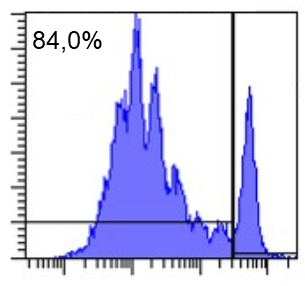 | 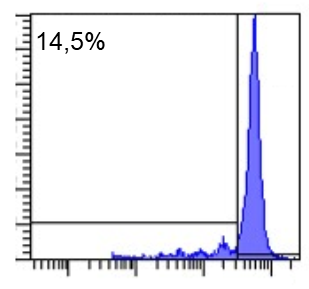 |
| **Secukinumab** | 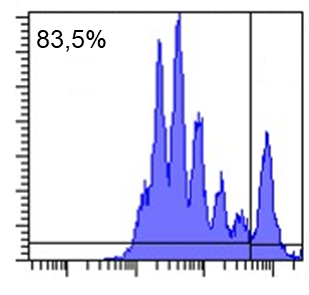 | 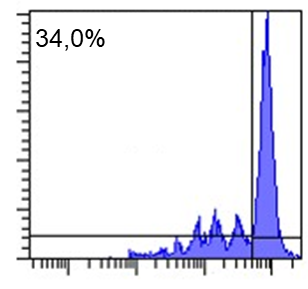 | 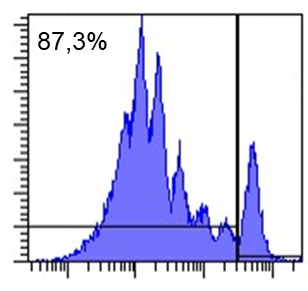 | 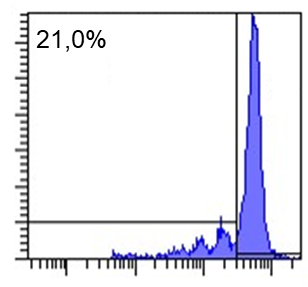 |

X-axis: CFSE (Carboxyfluorescein-succinimidyl-ester)-amount; Y-axis: cell count
Percentages show proliferated lymphocytes (left part of graph-cut-off) out of all CFSE-marked lymphocytes.
Suppressive function of Treg was measured by comparison of PBMC proliferation in Treg 0 : PBMC 1 co-culture with PBMC proliferation in Treg 1 : PBMC 1 co-culture.
